# Genomic Landscape of Actionable Mutations in Primary and Metastatic Tissues of Colon Adenocarcinoma

**DOI:** 10.7759/cureus.24175

**Published:** 2022-04-16

**Authors:** Emre Yekedüz, Hakan Akbulut, Güngör Utkan, Yüksel Ürün

**Affiliations:** 1 Medical Oncology, Ankara University School of Medicine, Ankara, TUR

**Keywords:** braf mutation, k-ras mutations, neoplasm metastasis, colorectal neoplasms, genomics

## Abstract

Aim

To assess the actionable genomic landscape of colon adenocarcinoma in the primary and metastatic tumor tissues.

Methods

The data from the American Association for Cancer Research (AACR) Project Genomics Evidence Neoplasia Information Exchange (GENIE) were used in this study. Colon adenocarcinoma patients with primary and metastatic tissue samples (distant organ and lymph node) were selected. Patients with samples from a local recurrence, not otherwise specified tumor samples, and data not collected for sampling localization were excluded.

Results

A total of 3286 and 1727 patients were included in the primary and metastatic tissue sample groups, respectively. There was no difference between the groups in Kirsten rat sarcoma viral oncogene homolog (KRAS) mutation rates. The rates of v-Raf murine sarcoma viral oncogene homolog B (BRAF) and mismatch repair (MMR) gene mutations were higher in the primary tumor tissues than in the metastatic tumor tissues. There was also no difference between the groups in other actionable gene alterations (e.g. ERBB2 amplification and neurotrophic receptor tyrosine kinase (NTRK) 1 and NTRK3 fusions). In contrast to all cohorts, in Asian and black patients, there was no difference in actionable genomic landscape between the primary and metastatic tumor tissues.

Conclusion

This study had the largest number of colon cancer patients that evaluated the actionable genomic alterations in primary and metastatic tumor tissues. BRAF and MMR gene alterations were more frequent in the primary tumor tissues than the metastatic tumor tissues.

## Introduction

Molecular analysis in metastatic colorectal cancer (CRC) patients is the mainstay of treatment decision-making. Kirsten rat sarcoma viral oncogene homolog (KRAS), neuroblastoma RAS viral oncogene homolog (NRAS), and v-Raf murine sarcoma viral oncogene homolog B (BRAF) mutations, human epidermal growth factor receptor 2 (HER2) (ERBB2) amplification, and neurotrophic receptor tyrosine kinase (NTRK) fusions are the actionable gene alterations in CRC patients. On the other hand, mismatch repair (MMR) enzyme deficiency caused by mutations in MMR genes is a predictive and prognostic factor, especially in the early stage of CRC. Thus, the National Comprehensive Cancer Network (NCCN) guidelines recommend evaluating these genomic alterations in CRC patients [1].

During the genomic evolution of CRC, which is an active process in tumor development, inter-tumor and intra-tumor heterogeneity may occur. As a result of this genomic evolution and tumor heterogeneity, genomic alterations may differ in the early and late stages of cancer [2]. In this regard, primary and metastatic tissues may also present different mutational landscapes due to the intra-tumoral heterogeneity [3,4]. In addition to intra-tumor heterogeneity, CRC has various genomic alterations depending on tumor location. For instance, BRAF mutations and microsatellite instability (MSI) were more frequent in right-sided tumors [5].

Genomic heterogeneity in CRC is not only a prognostic factor but it may also affect treatment outcomes with chemotherapy and targeted therapy agents. In this context, during or after treatment, the selection of some subclones having an advantage due to their genomic alterations may cause drug resistance [6,7].

To the best of our knowledge, up to now, there were studies with a small number of patients and a meta-analysis that assessed the primary and metastatic tumor tissues for genomic alterations in CRC [8]. This study aimed to assess and compare the primary and metastatic colon cancer tissues for actionable mutations by using the American Association for Cancer Research (AACR) Project Genomics Evidence Neoplasia Information Exchange (GENIE) [9].

## Materials and methods

The AACR Project GENIE

In this study, we used data from the AACR Project GENIE [10]. The AACR Project GENIE is a multi-institutional and international cancer genomic data-sharing program. We used the version 8.1 cohort. About 90,000 patients were included in this version [9]. Institutional board review approvals and informed consent were received by the centers. All laboratories have Clinical Laboratory Improvement Amendments (CLIA)/International Organization for Standardization (ISO) certificates [10]. The genomic data include mutations, copy number alterations, and structural abnormalities. All genomic analyses are carried out via next-generation sequencing techniques [10]. Detailed information about the centers and procedure of genomic analyses can be accessed on the AACR Project GENIE's website [9].

Patients cohort and data extraction

We extracted data from the GENIE project version 8.1 cohort. To access the data, we used cBioPortal for cancer genomics (genie.cbioportal.org). We selected colon adenocarcinoma patients through OncoTree cancer type taxonomy. After selecting colon adenocarcinoma patients, we categorized patients into the following two groups: patients with primary cancer tissue samples and patients with metastatic cancer tissue (distant organ and lymph node) samples. Patients with samples from a local recurrence, not otherwise specified tumor samples, and data not collected for sampling localization were excluded from the study. Finally, we included 3286 and 1727 patients with 3405 and 1866 samples in the primary and metastatic tumor tissue groups, respectively. For concordance analysis, patients with both primary and metastatic tumor tissues were selected from the database.

We extracted baseline characteristics (e.g. age at sampling, gender, race, and mutation count in each patient) and genomic data (e.g. mutations, copy number alterations, and structural abnormalities) to a database. We evaluated only actionable colon adenocarcinoma gene alterations (e.g. KRAS, NRAS, and BRAF mutations, ERBB2 amplification, and NTRK1 and NTRK3 fusions) in this study.

Statistical analysis

We used the median with an interquartile range (IQR) for continuous variables and percentages for categorical variables in the descriptive analyses. To compare categorical variables, we used chi-square or Fisher’s exact test. P-values corrected by the Benjamini-Hochberg method were used to determine q-values. A q-value of less than 0.05 was accepted as statistically significant. We also conducted Cohen’s kappa analysis for concordance analysis. SPSS version 27.0 for Mac (IBM Corp., Armonk, NY) was used for statistical analyses.

## Results

A total of 5183 patients were included in this study. The total number of samples was 5356 and 173 patients had more than one tissue sample. KRAS mutations were the most common genomic alteration among the involved genes, and 41.4% of all patients had KRAS mutations. Besides, BRAF mutations were observed in 13.3% of all patients. MSH6 was the most frequent mutant MMR gene. Frequencies of all included genes are shown in Table 1.

**Table 1:** Gene alteration frequencies in colon adenocarcinoma cohort MAPK: mitogen-activated protein kinase; KRAS: Kirsten rat sarcoma viral oncogene homolog; NRAS: neuroblastoma RAS viral oncogene homolog; BRAF: v-Raf murine sarcoma viral oncogene homolog B; NTRK: neurotrophic receptor tyrosine kinase.

	Patients, n = 5183 (%)	Samples, n = 5356 (%)
MAPK pathway		
KRAS	2247 (41.4)	2308 (43.1)
NRAS	251 (4.8)	255 (4.9)
BRAF	694 (13.3)	712 (13.7)
Mismatch repair genes		
MLH1	138 (2.7)	138 (2.6)
MSH2	144 (2.8)	137 (2.5)
MSH6	213 (4.1)	212 (3.9)
PMS2	91 (1.7)	91 (1.6)
ERBB2 amplification	75 (1.4)	79 (1.4)
NTRK fusions		
NTRK1	10 (0.2)	12 (0.2)
NTRK3	6 (0.1)	6 (0.1)

Comparison of primary and metastatic tissues

A total of 3405 (63.5%) tissues were obtained from the primary tumors, while the remaining tissues (n = 1866, 36.5%) were obtained from the metastatic tumor sites. The median age at sampling was 59 (IQR: 49-68) and 58 years (IQR: 49-67 years) in the primary and metastatic sampling groups, respectively. Of patients, 52% and 54% were males in the primary and metastatic sampling groups, respectively. About 70% of patients were white in each group. Baseline characteristics of included patients and centers are shown in Table 2.

**Table 2:** Baseline characteristics in primary and metastatic sample groups IQR: interquartile range.

	Primary, n = 3405 (%)	Metastasis, n = 1866 (%)
Age at sampling, median (IQR)	59 (49-68)	58 (49-67)
Mutation count, median (IQR)	7 (4-11)	6 (3-9)
Gender, male	1731 (52)	954 (54)
Race		
White	2432 (73.4)	1221 (69.1)
Black	253 (7.6)	137 (7.7)
Asian	166 (5.1)	71 (4.1)
Centers		
Memorial Sloan Kettering Cancer Center	1314 (38.5)	844 (45.2)
Dana-Farber Cancer Institute	962 (28.2)	313 (16.7)
Johns Hopkins Sidney Kimmel Comprehensive Cancer Center	381 (11.1)	167 (8.9)
Princess Margaret Cancer Centre	153 (4.4)	74 (3.9)
Vanderbilt-Ingram Cancer Center	123 (3.6)	19 (1.1)
Herbert Irving Comprehensive Cancer Center	120 (3.5)	113 (6.1)
Netherlands Cancer Institute	96 (2.8)	91 (4.3)
Duke Cancer Institute	81 (2.3)	91 (4.3)
Vall d’Hebron Institute of Oncology	56 (1.6)	34 (1.8)
Swedish Cancer Institute	41 (1.2)	26 (1.3)
University of Chicago Comprehensive Cancer Center	35 (1.1)	11 (0.5)
Wake Forest University Health Sciences	21 (0.6)	14 (0.7)
Yale University (Yale Cancer Center)	19 (0.5)	25 (1.3)
Institut Gustave Roussy	2 (0.1)	54 (2.8)
Providence Health & Services, Cancer Institute	1 (0.05)	0

A total of 3286 and 1727 patients were included in the primary and metastatic tissue sampling groups, respectively. KRAS mutations were the most common interested genomic alterations in each group, and there was no difference between the groups (43.9% and 43.5% in primary and metastatic groups, q = 0.6). BRAF mutations were higher in the primary tissue sampling group than in the metastatic tissue sampling group (15.4% and 9.5% in the primary and metastatic groups, q < 0.001). Similarly, MMR gene mutations except PMS2 mutation were higher in the primary tissue sampling group than in the metastatic tissue sampling group, and the difference between the groups was statistically significant. Additionally, there was no difference between the groups in ERBB2 amplification and NTRK1 and NTRK3 fusions (Table 3).

**Table 3:** Comparison of primary and metastatic tissues for actionable gene alterations MAPK: mitogen-activated protein kinase; KRAS: Kirsten rat sarcoma viral oncogene homolog; NRAS: neuroblastoma RAS viral oncogene homolog; BRAF: v-Raf murine sarcoma viral oncogene homolog B; NTRK: neurotrophic receptor tyrosine kinase.

	Primary, n = 3286 (%)	Metastasis, n = 1727 (%)	q
MAPK pathway			
KRAS	1483 (43.9)	791 (43.5)	0.6
NRAS	152 (4.5)	94 (5.1)	0.3
BRAF	522 (15.4)	173 (9.5)	<0.001
Mismatch repair genes			
MLH1	114 (3.4)	21 (1.1)	<0.001
MSH2	111 (4.2)	31 (2.2)	0.006
MSH6	167 (6.2)	41 (2.9)	<0.001
PMS2	69 (2.6)	22 (1.6)	0.1
ERBB2 amplification	46 (1.8)	36 (2.7)	0.5
NTRK fusions			
NTRK1	8 (0.3)	2 (0.1)	0.2
NTRK3	4 (0.1)	2 (0.1)	0.6

Subgroup analysis for gender and race

**Figure 1:**
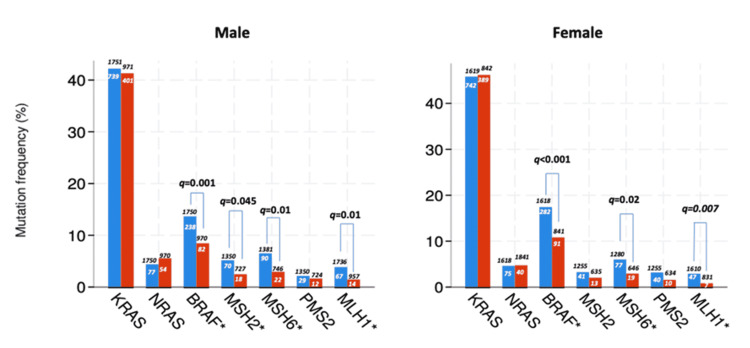
Genomic alterations in each gender Blue bars represent primary tumor tissue and red bars represent metastatic tumor tissue. Numbers inside the bars indicate patients with genomic alteration and numbers outside the bars indicate all patients with genomic analysis. * Difference between the groups was statistically significant. KRAS: Kirsten rat sarcoma viral oncogene homolog; NRAS: neuroblastoma RAS viral oncogene homolog; BRAF: v-Raf murine sarcoma viral oncogene homolog B.

We performed a subgroup analysis for gender and race to compare the genomic alteration rates in the primary and metastatic tumor tissues. There was no difference between the male and female patients in ERBB2 amplification rates. However, we did not evaluate NTRK 1 and NTRK 3 fusions due to the low number of patients to compare. BRAF mutations were higher in the primary tumor tissues than the metastatic tumor tissues in male and female patients. MMR gene alterations were similar to the whole cohort. However, in female patients, MSH2 mutation rates did not differ in the primary and metastatic tissues. Mutation frequency in each gender is shown in Figure 1.

**Figure 2:**
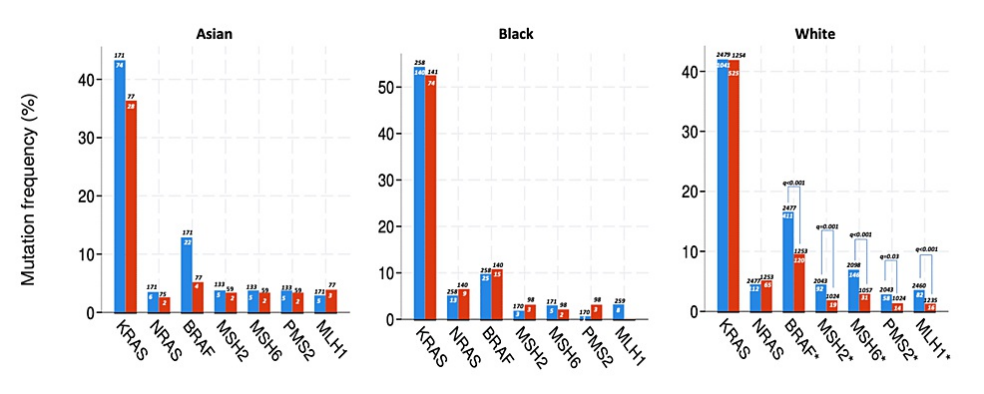
Genomic alterations in each race Blue bars represent primary tumor tissue and red bars represent metastatic tumor tissue. Numbers inside the bars indicate patients with genomic alteration and numbers outside the bars indicate all patients with genomic analysis. * Difference between the groups was statistically significant. KRAS: Kirsten rat sarcoma viral oncogene homolog; NRAS: neuroblastoma RAS viral oncogene homolog; BRAF: v-Raf murine sarcoma viral oncogene homolog B.

In white patients, the genomic landscape was similar to all cohorts. However, in Asian and black patients, there was no difference between the primary and metastatic tissues for genomic alterations. ERBB2 copy number alterations were similar in primary and metastatic tissues for each race. Mutation rates for each race are shown in Figure 2.

Concordance between the primary and metastatic tissues

We included 88 patients with paired primary and metastatic tissues. We assessed KRAS and BRAF mutations as the two most common genomic alterations among the included genes. Of patients, 36 and 37 had KRAS mutations in the primary and metastatic sampling groups, respectively. Three patients had KRAS wild-type (WT) tumor in the primary tissue and KRAS mutant (MT) tumor (two patients had KRASG12A and one patient had KRASG13D mutations) in the metastatic tissue. On the other hand, two patients had KRAS WT tumor in the metastatic tissue and KRAS MT tumor (one patient had KRASG13C and one patient had KRASG12D) in the primary tissue. Concordance between the primary and metastatic tissues was 88.4% (Cohen’s kappa).

Thirteen patients had BRAF mutations in each group. One patient had a BRAF WT tumor in the primary tissue and a BRAF MT (BRAFP277Hfs*2) tumor in the metastatic tissue. Similarly, one patient had a BRAF WT tumor in the metastatic tissue and a BRAF MT (BRAFN581H) tumor in the primary tissue. Concordance between the primary and metastatic tissues was 91% (Cohen’s kappa). The disposition of KRAS and BRAF mutations in each group is shown in Table 4.

**Table 4:** Concordance between the primary and metastatic tissues (n = 88) KRAS: Kirsten rat sarcoma viral oncogene homolog; BRAF: v-Raf murine sarcoma viral oncogene homolog B.

		Metastatic		Cohen’s kappa
		KRAS mutant, n (%)	KRAS wild, n (%)	88.4%
Primary	KRAS mutant	34 (91.8)	2 (3.9)	
	KRAS wild	3 (8.2)	49 (96.1)	
		Metastatic		
		BRAF mutant, n (%)	BRAF wild, n (%)	91%
Primary	BRAF mutant	12 (92.3)	1 (1.3)	
	BRAF wild	1 (7.7)	74 (98.7)	

## Discussion

To the best of our knowledge, this was the most comprehensive study, including the highest number of patients to compare the genomic alterations in primary and metastatic tumor tissues of colon adenocarcinoma. This study revealed that BRAF and MMR gene mutations were lower in the metastatic colon cancer tissues than in the primary tumor tissues. On the other hand, there was no difference in the rates of KRAS and NRAS mutations, ERBB2 amplification, and NTRK1 and NTRK3 fusions. Additionally, there was a very good concordance with the primary and metastatic colon adenocarcinoma tissues for KRAS and BRAF mutations.

It is well known that BRAF mutations usually accompany sporadic microsatellite instability-high (MSI-H) colon cancers. BRAF mutations were more frequent in right-sided colon cancers than the left-sided ones. Besides, MSI-H tumors were also more frequent in the right-sided colon cancers [11]. In this context, we revealed that BRAF mutations and MMR gene mutations were higher in the primary tumor tissues. There may be more than one reason for this outcome. BRAF mutant tumors may be less likely to have distant organ metastases. In a study, Tran et al. showed that BRAF mutations were associated with higher peritoneal and distant lymph node metastasis but not lung metastasis [12]. Similar to this result, Melloni et al. established that the pulmonary metastasis rate was lower in CRC patients with MSI-H tumors [13]. Tumor heterogeneity may also be accepted as a reason for the difference in genomic landscape between the primary and metastatic tumor tissues. Tumor heterogeneity originates from the subclones having distinct mutations in the same tumor. Adjuvant anti-cancer treatment may lead to the selection of some subclones, and these advantaged subclones may finally accelerate the recurrence and metastasis [14,15]. In this study, we do not have information as to whether metastatic tissues belong to patients with de novo metastasis or not. In this regard, metastasis after an adjuvant treatment might have led to selecting some subclones without BRAF and MMR gene mutations. Discordance of genomic alterations between the primary and metastatic tumor tissues might explain the different mutation types in those groups. However, in a subgroup of patients, we assessed the primary and paired metastatic tissues for KRAS and BRAF mutations. Similar to the previous studies, concordance was better in each gene [16]. Furthermore, 9% of discordance in the BRAF mutations originated from non-V600E BRAF mutations. BRAFV600E mutation is the most common BRAF mutation subtype in metastatic and early-stage CRC patients, and it is a bad prognostic factor in those patients [17-19]. Due to the bad prognosis, most of these patients may have unresectable metastases. Thus, metastasectomy rates may be lower in those patients. A low BRAF mutation rate in the metastatic tissues in our study may be associated with a higher rate of unresectable metastases.

Similar to the results of our study, a comprehensive meta-analysis evaluating the BRAF mutation rate in CRC patients showed that BRAF mutations were more frequent in the primary tissues than the metastatic tissues [8]. However, it should be noticed that it was not an individual patient-level data meta-analysis.

BRAF mutant CRC had a distinct pathology and clinical course. The BEACON trial showed the effectiveness of cetuximab plus encorafenib plus binimetinib in BRAF mutant metastatic CRC patients [20]. In contrast to previous studies, the BEACON trial concluded that triplet inhibition of epidermal growth factor receptor (EGFR), BRAF, and mitogen-activated protein kinase kinase (MEK) was associated with better outcomes than BRAF inhibition alone [20,21]. In our study, the BRAF mutation rate was 15.4% and 9.5% in the primary and metastatic tissues. There are no data for the use of BRAF inhibition in the adjuvant setting. However, stage II-III CRC patients with BRAFV600E mutation had worse outcomes. BRAF inhibition in the early stages of colon cancer may be considered a new therapeutic target in those patients. We need more results from clinical trials.

Racial diversity may affect the genomic landscape in tumorigenesis. In a study that compared the BRAF and KRAS mutations, Asians had fewer BRAF and KRAS mutations than white and black patients [22]. However, in the literature, there were no data for comparing primary and metastatic colon cancer tissues in different races. In our study, we revealed that primary and metastatic cancer tissues had a similar actionable genomic landscape in Asian and black patients in contrast to white patients. In white patients, BRAF and MMR gene mutations were higher in the primary tumor tissue than in the metastatic tumor tissue.

Our study has several limitations. First, the AACR GENIE project does not include the data for survival and cancer treatment. That is why we did not evaluate the impact of genomic alterations on the survival outcomes. In addition, we did not interpret the effect of adjuvant treatment on the tumor heterogeneity between the primary and metastatic tissues. Second, we did not assess the BRAF mutations in detail. In colon cancer patients, the majority of BRAF mutations involve BRAFV600E mutation. However, the other BRAF mutations can be observed in CRC patients. Furthermore, clinical course and prognosis are better in non-V600E BRAF mutant CRC patients [23]. Third, we included only two gene alterations in the concordance analysis. We did not assess NRAS mutation, ERBB2 amplification, and NTRK1 and NTRK3 fusions due to the scarce number of patients. Fourth, we did not have data for the tumor location in the primary tumors. It is well known that right-sided and left-sided tumors have different genomic alteration landscapes [24]. The proportion of samples from the right or left-sided tumors might have affected the results of our study.

## Conclusions

In conclusion, this study had the largest number of colon cancer patients that evaluated the actionable genomic alterations in primary and metastatic colon cancer tissues. BRAF and MMR gene alterations were more frequent in the primary tumor tissues than the metastatic tumor tissues.
